# Gold Nanoparticles with Adaptable Self-Assembled Monolayer Shells Allow Multivalent Inhibition and Sensing of Influenza Virus at Ultralow Concentrations

**DOI:** 10.1021/acscentsci.5c00602

**Published:** 2025-08-07

**Authors:** Yulia Sergeeva, Sing Yee Yeung, Thomas Hix-Janssens, Börje Sellergren

**Affiliations:** Department of Biomedical Sciences and Biofilms-Research Center for Biointerfaces (BRCB), Faculty of Health and Society, Malmö University, 205 06, Malmö, Sweden

## Abstract

Multivalent inhibitors that mimic the polysaccharide array on cells represent a new paradigm in the development of antiviral agents and antibiotics. Covalent ligand anchoring limits the affinity and, in turn, potency of these inhibitors with dissociation constants (*K*_d_) commonly found in the micromolar or upper nanomolar range. Addressing this deficiency we here report on easily accessible gold core–shell nanoparticles (rSAM-NPs) featuring adaptable reversible self-assembled monolayer (rSAM)-based shells. The rSAMs are anchored by noncovalent amidinium-carboxylate interactions on gold nanoparticles at slightly alkaline pH resulting in laterally mobile pH-responsive assemblies that are functional at physiological pH. Introducing sialic acid ligands in the shell, we show that the rSAM-NPs strongly interact with the influenza virus surface protein hemagglutinin (limit of detection LoD < 2 nM) and deactivated bird flu virus H5N1 (LoD < 1 HAU) in allantoic liquid. Finally, we show that the rSAM-NPs effectively inhibit the interaction of the virus with red blood cells at concentrations in the low picomolar range. This represents a significant increase in potency with respect to multivalent inhibitors of similar size based on covalently anchored monosaccharides.

## Graphical abstract

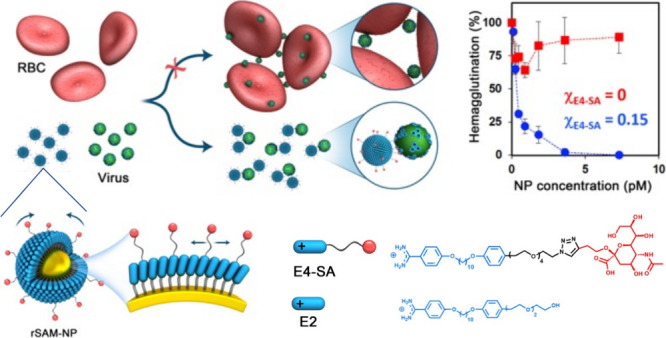

## Introduction

Multivalent interactions govern numerous biorecognition events at nanoscopic or microscopic length scales.^1–5^ Like a biological Velcro, these contacts rely on multiple individually weak but rapidly exchanging interactions between surface-confined receptors or ligands on the two interacting partners. One of the most ubiquitous multivalent interactions involves carbohydrates and lectins, where a single lectin’s intrinsically weak affinity for the carbohydrate (*K*_d_ ≈ 10^–3^–10^–6^ M) translates into an exceptionally strong and selective interaction when multiplied.^6–10^ These interactions govern cell and virus recognition in numerous key biological processes such as cell–cell communication, fertilization, biofilm formation, cell differentiation, cancer metastasis, viral infection, and bacterial adhesion.

As in low molecular inhibitor design, multivalent inhibitors have been designed to block or modulate the aforementioned interactions.^3,11–13^ For instance, ligand decorated polymers,^14–16^ nanoparticles,^17–21^ or dendrimers^22^ have been reported to display a high affinity for cell surface receptors. These systems provide a globular homogeneous ligand array that mimics the carbohydrate representation on the cell surface and allows control over the ligand’s chemical composition, ligand density, spatial arrangements, and accessibility. Careful tuning of the carbohydrate separation distance to match the lectin binding site separation and adjustments of the carbohydrate presentation and orientation have led to particularly powerful binders and inhibitors.^13,18–20,22–24^ Such scaffolds can interrupt pathogen-host cell interactions and represent an attractive target for the proper design of new antibacterial or antiviral drug candidates.^7,11–13,15,18^

Despite the aforementioned advances, the majority of multivalent inhibitor designs exhibit only modest affinity enhancements, as exemplified by monosaccharide based multivalent inhibitors with *K*_d_’s in the micromolar range. One reason for this is their failure to mimic the dynamics and adaptable nature of the biological interactions. To compensate for this deficiency, a challenging Ångstrom precise tuning of ligand separation distance and presentation (minimizing conformational and rotational loss of entropy upon binding) is required to boost inhibitor affinity albeit at the expense of demanding synthetic procedures.^13^ To overcome this problem the use of supported lipid bilayers (SLBs) or liposomes exhibiting mobile ligands that can diffuse laterally to optimize receptor binding is an attractive approach for generating strong multivalent interactions.^5,10,25^ This mimics the lateral diffusion of receptors and ligands occurring in cell membranes, a feature cleverly used by nature to promote so-called “superselective” multivalent interactions using only a few receptor copies.^9^ In this sense, bioactive ligand-functionalized vesicles or membrane covered nanoparticles hold promise as broad spectrum biocompatible virus inhibitors.^26–29^ These are based on core–shell nanoparticles with a solid core surrounded by a fluidic lipid bilayer shell made by using the plasma membranes of a specific cell type and have shown impressive potency in blocking viral infections.

As an abiotic alternative to such systems, we here introduce core–shell nanoparticles (rSAM-NPs) featuring reversible self-assembled monolayer (rSAM) shells and show how they can be used as a platform to construct highly potent multivalent virus inhibitors (Figure 1). rSAMs offer robust and adaptable receptors featuring strongly enhanced affinities toward proteins,^30^ viruses,^31,32^ and human cells^33^ compared to receptors based on stiffer scaffolds. The formation of the rSAMs is driven by the spontaneous assembly of benzamidine-terminated amphiphiles on alkanoic acid functionalized surfaces (e.g., SAMs of thiols on gold) in a neutral or alkaline aqueous solution.^34–36^ This leads to monolayers featuring thicknesses correlating with the molecular length of the amphiphile accompanied by a lipid bilayer-like lateral diffusivity of the layer components.^30^ In contrast to lipid-based models, the benzamidine monolayer is pH-responsive, resistant to plasma protein exchange, and stable to rinsing and air exposure. Based on this reversible sensor platform we have recently constructed glycan-based sensors for influenza virus^31^ and lectins^30^ featuring picomolar detection limits. We ascribed the high sensitivity to laterally mobile sialic acid ligands, promoting superselective multivalent interactions. This leads to affinities much exceeding the affinity of covalent sensor constructs.^31^

**Figure 1:**
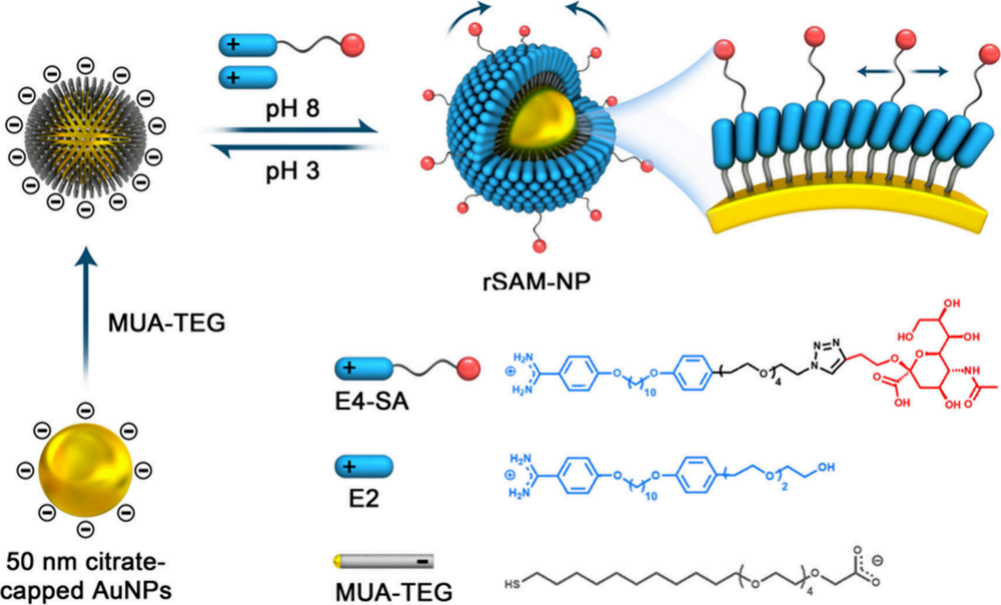
Schematic illustration of the preparation of sialic acid functionalized rSAM-NPs.

We describe in this report the design of rSAM-NPs and demonstrate their ability to bind low levels of H5N1, lectins and virus-like particles. Finally, we show how these scaffolds can interrupt virus-host cell interactions at ultralow concentrations, significantly lower than those of previously reported glyconanomaterial-based inhibitors. Based on our findings, we believe this dynamic ligand construct to represent a competitive abiotic alternative to high affinity lipid-based receptors and pathogen inhibitors.

## Results and Discussion

### System Design and Characterization

The design of multivalent ligand architectures for virus inhibition requires attention to the structural features of the receptor in terms of ligand receptor binding affinity and specificity, receptor valency, site separation, virus size, and virus mechanical properties.^3,9,10^ Influenza A type viruses (IAV) are pleomorphic enveloped RNA viruses with diameters of 100–150 nm containing 290–340 hemagglutinin (HA) trimers and 24–50 neuraminidase (NA) tetramers on their surface. The SA binding site separation has been estimated to ca. 4 nm within the HA trimer and on average 12 nm between individual HA trimers. Infection is initiated by the virus attaching to the host cell through formation of multiple (estimated to ca. 10–20 per particle) weak (*K*_d_ ≈ 10^–3^ M) interactions between HA and abundant cell surface sialic acids (SA), the latter modulated by the SA cleaving enzyme NA. Use of fluidic models of the host cell membrane has demonstrated a strong dependence of virus adhesion on SA surface coverage and tether length with an onset of virus binding occurring at an SA coverage of 1–5 pmol/cm^2^ beyond which superselective virus binding is seen.^5,9^ This coverage corresponds to an approximate SA-lipid/lipid ratio of 1–2%.

The key parameters investigated to optimize avidity for this virus have therefore been the density of ligands, linker length and rigidity, nature of scaffold and its rigidity/fluidity, as well as the contact area between virus and inhibitor.^3,7,13,15^ Knowledge based on other host cell receptor models and inhibitor designs made us reason as follows for the rational design of rSAM based inhibitors.

#### Nature of the Scaffold

Gold nanoparticles (AuNPs) have been extensively investigated as core for immobilizing appropriate ligands in a controlled manner exploiting the ease of synthesis and precisely controlled surface chemistry achievable in SAMs of thiols on gold.^37,38^ Moreover AuNPs are biocompatible scaffolds of tunable size and optical properties with a surface plasmon band position and width strongly influenced by the gold core size and the nature of ligands on its surface. The latter also influence their colloidal stability in addition to their concentration, pH, and ionic strength. AuNPs have also been used as scaffolds for multivalent pathogen inhibition.^13,17,18^ In one notable example, multivalent inhibition of hemagglutination in the nanomolar range was demonstrated using hyperbranched SA terminated dendron-coated AuNPs.^17^ As a logical extension of our previously demonstrated 2D rSAM based IAV receptors using planar gold^31^ we wanted to explore whether this chemistry could be transferred to 3D in the form of rSAM modified gold nanoparticles. This was inspired by our previous findings that bisbenzamidines interact strongly and rapidly precipitate negatively charged carboxylated AuNPs.^39^ The particle size is expected to strongly influence the inhibitor performance. Since the number of ligand–receptor pairs increases with the contact area, a tighter binding has been found for size matched inhibitors.^16^ We therefore decided to investigate 50 and 100 nm gold as carriers for the rSAM shell, which we anticipated would lead to sizes in the same range as the IAV particles (100–150 nm). Also, we included differently shaped particles (rods and cubes) to account for the pleomorphic nature of the IAV particles. To optimize colloidal stability of the NPs in aqueous media we decided to perform ligand exchange of the citric acid stabilized particles with mercaptoundecane-tetraethylenglycol-based (MUA-TEG) carboxylic acid thiol (Figure 1) as it combines a long hydrophobic aliphatic chain promoting the formation of an ordered SAM, and a short oligo­(ethylene glycol) spacer that increases the NP colloidal stability in aqueous media and reduces nonspecific binding. The carboxylic headgroups allow interaction with the amidinium ion, promoting the formation of the reversible self-assembled monolayers (rSAMs).

#### Ligand Density and Presentation

The minimum ligand density required for superselective virus binding depends on whether the ligands are attached to static or fluidic scaffolds and the length of the linker connecting the SA with the scaffold.^5,9^ As discussed above, the density in fluidic scaffolds typically shows superselective binding at low ligand densities, i.e. at 1–2% ligand modified lipids or even lower using SLB scaffolds, whereas static scaffolds require higher densities. With regard to the linker length, long linkers allow the SA ligands to better access the lectin binding site further reducing the ligand density onset for superselective binding. We previously showed that mixed rSAMs of the SA functionalized amidines (Figure 1) and filler amidines display strongly enhanced affinity for HA and VLPs at SA coverages of 15 mol % (χ_E4‑SA_ = 0.15) and linkers containing 4 ethylene glycol repeat units such as in E4-SA (Figure 1).^30,31^ In contrast, rSAMs of E2 lacking E4-SA or with slightly increased E4-SA densities (χ_E4‑SA_ = 0.20) led to a complete suppression of binding, the latter ascribed to a paralel abrupt drop in ligand lateral diffusivity. As a starting point, we therefore decided to compare these rSAM compositions and whether similar effects would be observed for the 3D format.

### rSAM-Modification Results in Stable Colloids of Ligand Decorated Nanoparticles

To first confirm formation, structure, and properties of the films on planar substrates, we used in situ ellipsometry (ISE) and infrared reflection absorption spectroscopy (IRAS). Figure 2a shows the average film thickness during adsorption of a mixture of E2 and E4-SA (χ_E4‑SA_ = 0.15) on a SAM of MUA-TEG and Figure S1 the corresponding IRAS spectra after rinse with two different buffers. The rapid adsorption kinetics, the limiting film thickness, and the rinse stability (Figure 2a) are indicative of well-ordered films of densely packed amphiphiles oriented perpendicularly to the surface.^30^ Figure S1 and Table S1 support this picture revealing the significant vibrations with orthogonal transition dipole vectors of the anchor SAM (MUA-TEG) and the two component rSAMs as we reported previously.

**Figure 2:**
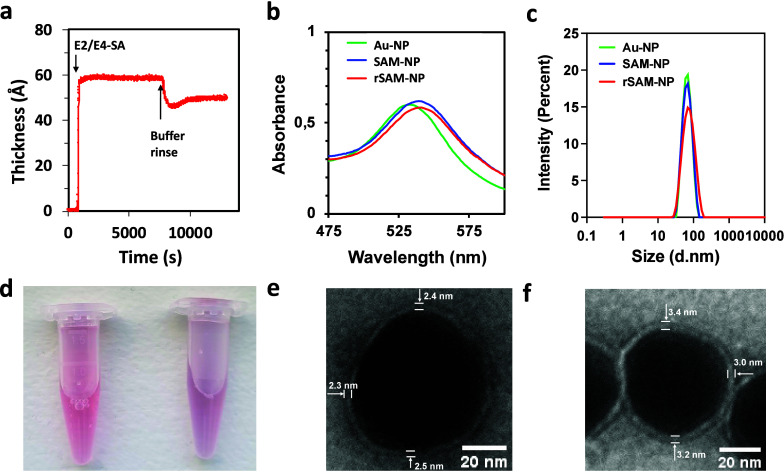
(a) Film thickness estimated by *in situ* ellipsometry versus time upon addition of a mixture of E2 and E4-SA (χ_E4‑SA_ = 0.15, *c* = 50 μM) in HEPES buffer (10 mM, pH 8) to MUA-TEG SAMs on gold. Final thicknesses measured after rinsing in HEPES pH 8-buffer were respectively 50 and 51 Å. (b) UV–vis absorption spectra of AuNPs (green line) after functionalization with MUA-TEG (blue line) and mixed rSAMs of E2 and E4-SA (χ_E4‑SA_ = 0.15) (red line) and (c) corresponding particle size distribution by DLS. (d) Picture of the SAM-NP (left) and rSAM-NP (right) colloidal solutions. (e, f) Transmission electron micrographs of 50 nm Au-NPs functionalized with MUA-TEG (e) followed by an rSAM of E2 and E4-SA (χ_E4‑SA_ = 0.15) (f). The estimated shell thicknesses were 2.4 ± 0.1 nm and 3.2 ± 0.2 nm, respectively.

The functionalization of the nanoparticles was then carried out in two steps, the success of each step being verified by DLS, FTIR, MALDI-TOF-MS, HPLC, and UV–vis spectroscopy (Figure 2b,c; Figure S2–S4; Table S2–S5). First, the citrate stabilized nanoparticles were functionalized with MUA-TEG via ligand exchange. This led to a 4 nm increase in the hydrodynamic diameter and a pronounced redshift (Δλ = 5 nm) of the surface plasmon band (Table 1; Table S2) manifested in a color change from red to bourdaux. The subsequent attachment of the amidines induced an additional red shift in the plasmon peak position of about 2 nm, resulting in a change in color of the colloidal solution from bordaux to purple (Figure 2b,d).

**Table 1:** Plasmon Peak Position and Hydrodynamic Size of the AuNPs in HEPES Buffer (10 mM, pH 8) before and after Functionalization with MUA-TEG (SAM-NPs) and E2/E4-SA (χE4-SA = 0.15) (rSAM-NP)^a^

ligand	λ (nm)	*D*_h_ (nm)	ζ-potential (mV)
Au-NP	533 ± 2	67 ± 2	–24 ± 4
SAM-NP	538 ± 0.3	71 ± 1	–40 ± 2
rSAM-NP	540 ± 0.4	73 ± 1	–25 ± 1

a Values are expressed as means ± SD, *n* = 3.

These particles formed stable colloids with no signs of agglomeration. The particle surface charge density reflected in the Z-potential (Table 1) decreased for these particles from −40 for the SAM-NPs to −25 after rSAM modification. This shows that the negatively charged carboxylate anchor SAM is partially neutralized by the rSAM amidinium counterions, the extent of which is difficult to estimate due to the negatively charged SA-ligand of the rSAM (p*K*_a_ (SA) = 2.6).

To quantify the amount of amidine amphiphiles adsorbed to the SAM-NPs we measured the amidine concentration by HPLC before and after incubation and calculated the uptake of each amidine from the depletion value (Table S3). As shown by the measured values corresponding to the total surface coverage (58%) and the incorporation of E4-SA (χ_E4‑SA_^HPLC^ = 0.3), amidine adsorption displayed a bias for E4-SA and failed to reach 100% site occupancy. These deviations from a random process are not unexpected given the known influence of surface curvature on packing density and the relative adsorption of charged and nonionic surfactants. More curvature often favors adsorption of charged surfactants with bulky head groups.^40,41^

To confirm the presence of the rSAM shell, we then characterized the rSAM-NPs by mass and infrared spectroscopy. MALDI-TOF-MS was used, as it features a mild ionization technique allowing analysis of neat samples of the NPs. Figure S3 shows a representative MALDI-TOF-MS spectrum of rSAM-NPs and the masses of the amphiphile molecular ions. The identification of signals arising from E2 at 501 *m*/*z* and E4-SA at 975 *m*/*z* provides evidence for the incorporation of both amphiphiles in the ligand shell.

FTIR-spectroscopy can be used to derive information regarding both the composition and molecular conformation of alkanethiol stabilized AuNPs.^42^ Although the FTIR band positions of the SAMs on such curved surfaces are similar to the IRAS spectra of planar SAMs, the bands are typically broader and lack the orientation-sensitive intensity dependence seen in IRAS. This is confirmed by Figure S4 showing the normalized attenuated total reflection infrared spectra (ATR-IR) of the SAM- and rSAM-NPs deposited on the ATR crystal from a pH 8 buffered solution. Noting here in brief that the rSAM modification results in the appearance of bands at 1620, 1273, and 838 cm^–1^ (for assignements see Table S3) that reasonably well match the rSAM spectral signature and a pronounced shift of the C–O–C ether stretch to lower frequencies (from 1186 to 1178 cm^–1^ upon rSAM modification) that we attribute to a conformational change of the TEG block, we refer to the Supporting Information for a more detailed spectral interpretation.

To finally visualize the NP architectures, we analyzed the particles by transmission electron microscopy (TEM) (Figure 2e,f). The samples were stained with uranyl acetate to determine whether the self-assembled coatings could be observed and to estimate their thickness. The average thickness of the MUA-TEG shell before (2.4 ± 0.1 nm) and after assembly of the rSAM shell (3.2 ± 0.2 nm) agreed with the data obtained by DLS (Tables 1, S2) and the coverage data derived by HPLC. Collectively, the above results support the presence of the rSAM on the MUA-TEG modified NPs.

### rSAM-NPs Interact Strongly and Selectively with Viral Proteins

To explore the affinity of the prepared rSAM-NPs for IAV, we first investigated their interactions with hemagglutinin (HA) from avian H5N1 (Table S6) by UV–vis spectroscopy and DLS. Assessing the plasmon band shift dependence of the AuNP core size and buffer medium showed that the use of smaller 50 nm AuNPs and phosphate buffer led to the strongest shifts (Figure S2, Figure S5). Hence, we fixed these parameters in all further experimentation. Selectivity was assessed using the α-mannose/α-glucose-binding lectin Concanavalin A (ConA) as a reference with the anchor NPs functionalized with MUA-TEG used as a control. The NPs were incubated with different concentrations of the proteins (0–12 nM), and the changes in the absorption spectra and the size of the nanoparticles were recorded 1 h after protein addition (Figure 3).

**Figure 3:**
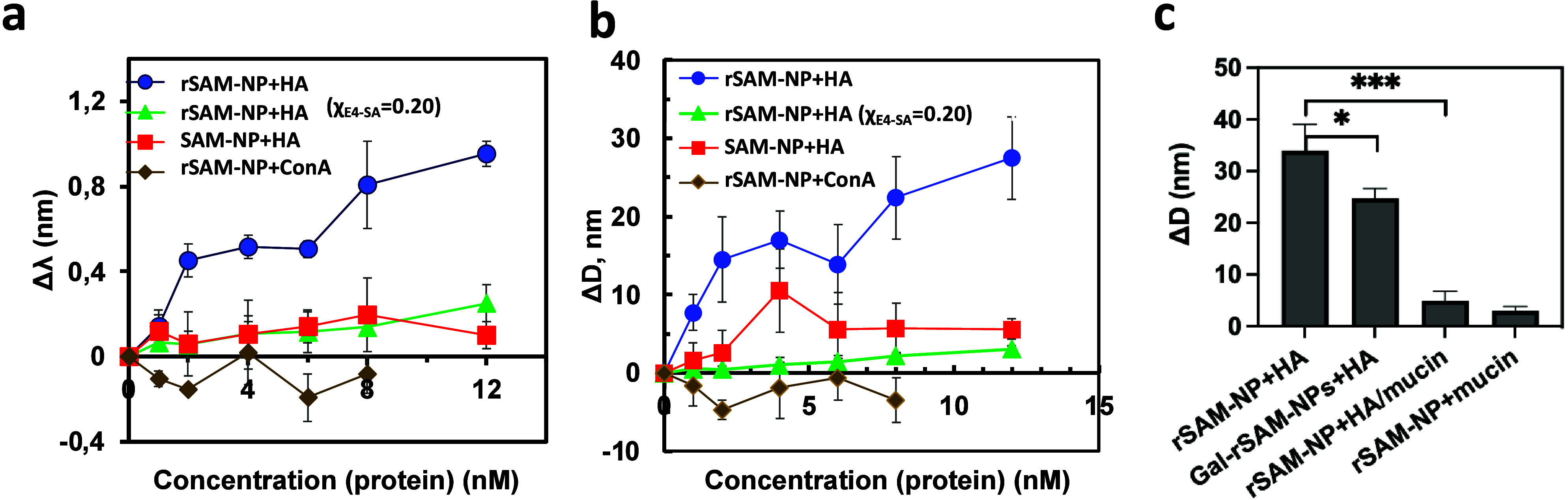
(a) Plasmon band shift of the rSAM-NPs (*c*_NP_ = 30 pM; χ_E4‑SA_ = 0.15: blue circle, brown diamond; χ_E4‑SA_ = 0.20: green triangle) and the anchor SAM-NP (red square) as a function of protein concentration in phosphate buffer (pH 7.4) upon the incubation with HA or ConA and (b) the parallel increase in the hydrodynamic diameter of the NPs. (c) The change in hydrodynamic diameter of rSAM-NPs and Gal-rSAM-NPs after the addition of HA (12 nM), a mixture of HA and mucin, or mucin alone (0.02 mg/mL). All values are expressed as means ± SD, *n* = 3.

The shift in the peak position was calculated by fitting the plasmon peak with the pseudoVoigt function using at least 40 data points. As shown in Figure 3a, the interaction with HA induced a gradual red shift in the SPR peak position of the rSAM-NPs with a limit of detection <2 nM. In contrast, only minor shifts were observed using the MUA-TEG based SAM-NPs. Moreover, no significant SPR and DLS shifts were observed for the reference protein ConA. These results were consistent with the data obtained by DLS (Figure 3b) revealing a pronounced size increase of the rSAM-NPs after incubation with HA. To further corroborate the selectivity of the interaction, we performed three additional control experiments: HA inhibition by mucin and ligand exchange. Mucin is an epithelial glycoprotein abundant in sialic acids. One of its roles is to act as a virus barrier by binding with high affinity (*K*_i_ = 2 × 10^–6^ M) to HA.^15^ Due to this inhibitory function, it can be used to probe the nature of the ligand receptor interactions. Figure 3c and Figure S6 show the size increase and the corresponding plasmon band shifts respectively of rSAM-NPs after exposure to HA, a mixture of HA and mucin or mucin alone. In agreement with our recent findings, preincubation of HA with mucin nearly completely suppressed the size increase and red shift observed in the absence of mucin, all in line with its role as an effective multivalent inhibitor. Moreover, mucin alone bound only weakly to the rSAM-NPs. Somewhat weaker but significant effects were observed when comparing SA and Gal functionlized rSAMs. Using the noncomplementary sugar Gal as a ligand led to a 10 nm smaller size increase compared to the SA ligand upon exposure to HA. Finally, we probed the NPs with the betacorona virus SARS-CoV-2 spike (S) protein, a trimeric surface protein which uses host cell sialylated glycans as coreceptors in variant dependent manner.^43^ Compared to the HA-SA interaction, binding has been shown to be much weaker (*K*_d_ ≫ 1 μM) and transient, most likely owing to the lack of neuraminidase activity found in IAVs. In agreement with these findings, our titrations (Figure S7) revealed significant LSPR red shifts and size increases but at ca. 10× higher concentrations.

Collectively, these data show that the rSAM-NPs, as intended, interact tightly with IAV lectin through the exposed SA ligands. Interestingly, the increase of the nominal E4-SA concentration to 20 mol % (χ_E4‑SA_ = 0.2) led to a drop of the HA binding (Figure 3a). Although not directly comparable with rSAMs on planar substrates (*vide supra*), this SA-density dependent inhibition is consistent with our recently reported data.^30^

The affinity of the rSAM- modified NPs for HA was further investigated in a depletion experiment using highly sensitive ELISA-based detection (Figure S8). To assess the impact of particle shape on HA binding we compared the spherical NPs with nanorods (NRs) and nanocubes (NCs) and incubated the particles in a 30 pM HA solution. Even at the lowest particle concentration (<10 pM) a more than 50% reduction in free HA was observed although with no obvious difference between the differently shaped AuNPs. This again indicates an exceptionally high affinity of the rSAM-NPs for the lectin.

### rSAM-NPs Interact Strongly with Deactivated H5N1

Next, we studied the interaction of the rSAM-NPs with the whole influenza virus in allantoic liquid, a protein and salt rich liquid originating from fetal membranes and used as a medium for virus production. This would test whether the shell architecture would survive in more competitive environments. For this purpose, rSAM-NPs were incubated for 1 h with solutions of inactivated H5N1 (A/Anhui/01/2005 H5N1; stock solution: 128 HAU, Figure S9), and then the samples were characterized by UV–vis spectroscopy (Figure 4) and DLS (Figure 5). Increasing the virus concentration led to clear changes in both the visual appearance of the solutions (Figure 4a) and their UV–vis spectra (Figure 4b). The plasmon band red-shifted, broadened, and decreased in intensity, while the absorbance at higher wavelengths increased. These changes indicate the presence of strong interactions between the virus and the particles and the formation of larger agglomerates.^44^ The shift of the plasmon band increased along with the virus concentration and appeared to level off at an IAV concentration of ca. 3 HAU with significant shifts observed for IAV concentrations as low as 1 HAU (Figure 4c).

**Figure 4:**
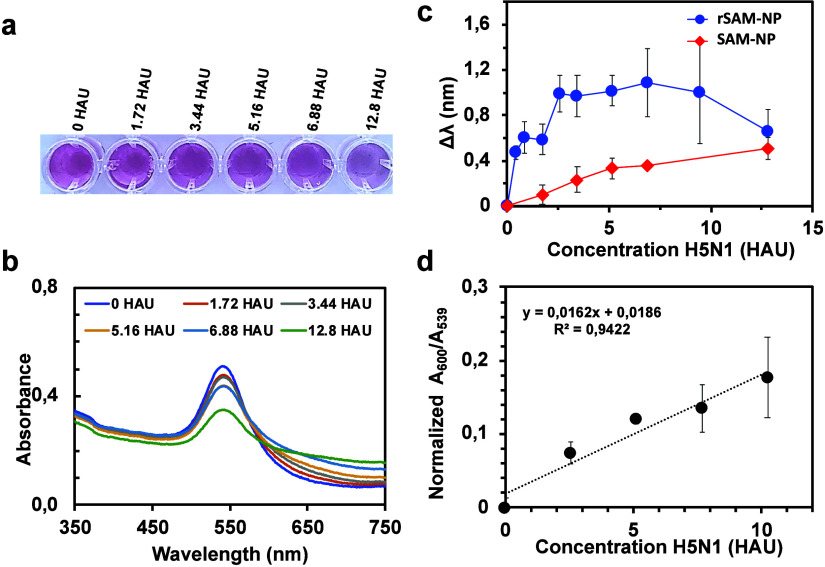
Top picture of the well plate (a) and corresponding UV–vis spectra (b) after 1 h incubation of the rSAM-NP colloidal solution (*c*_NP_ = 30 pM) with increasing concentrations of BPL-inactivated H5N1 in phosphate buffer (pH 7.4). (c) Plasmon band shifts after incubation of rSAM- and SAM-NPs. (d) Normalized *A*_600_ and *A*_539_ values of incubation of rSAMs NPs with inactivated H5N1. Values are expressed as means ± SD, *n* = 3.

**Figure 5:**
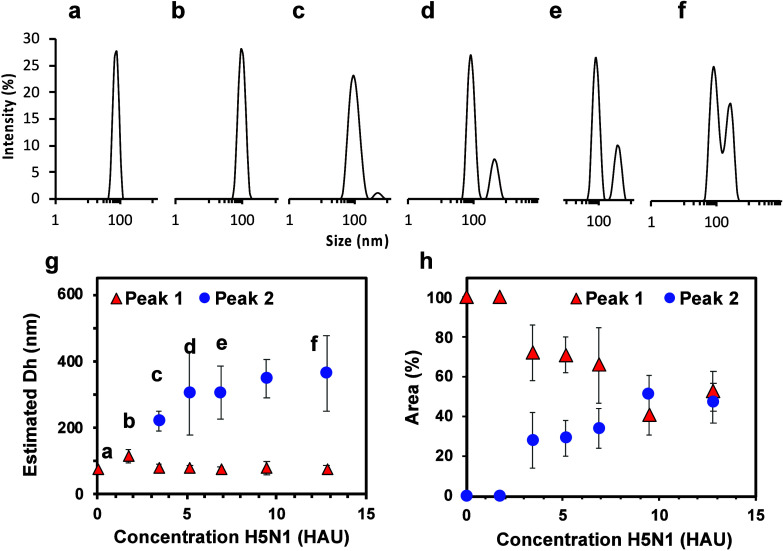
Size distribution analysis by DLS of the rSAM-NPs in PB (10 mM, pH 7.4, 0.005% Tween20) (a) and the NPs incubated with H5N1 at 1.72 HAU (b), 3.44 HAU (c), 5.16 HAU (d), 6.88 HAU (e), and 12.8 HAU (f). (g) Evaluation of the hydrodynamic diameter of rSAMs NPs and their agglomerates as a function of applied virus concentration. The letters indicating the corresponding size distribution curves have been included. (h) Peak area of peak 1 and peak 2 calculated from the DLS data analysis.

In view of the weak shifts observed for the SAM-NPs we conclude that these high-affinity interactions can be ascribed to the SA functionalized rSAM shell. For IAV concentrations exceeding 7.7 HAU, the plasmon band shift for the rSAM-NPs decreased, whereas the apparent measurement error increased. We assigned this phenomenon to the hook effect, a known limitation of immunoassays manifested in a decrease in the measured signal at high analyte concentrations.^45^ At elevated IAV concentrations, the binding sites on the NPs become saturated, which produces an increase in the interparticle distance and hence a decreased interparticle coupling. We therefore attribute the decreasing band shifts to this effect.^46^

The formation of agglomerates can be assessed by plotting the normalized *A*_600_/*A*_539_ ratio versus the concentration of the virus in suspension (Figure 4d).^44^ These data showed a quasi-linear correlation between the degree of rSAM-NPs aggregation and the virus concentration even at high HAU values. DLS confirmed this effect as shown by an additional population of particles of a size ranging from 200 to 450 nm appearing at virus concentrations above 5 HAU (Figure 5). Increasing the IAV concentration led to a further increase of the cluster peak area, reflecting a concomitant increase in the cluster concentration in the sample. In parallel, the area of the peak attributed to the individual rSAM-NPs decreased, reflecting the transition from well-dispersed NPs to agglomerates. All in all, these data show that the rSAM shell imparts a very high affinity for the targeted virus which exceeds most previously published multivalent platforms.^18,20^

### rSAM-NPs Effectively Inhibit Virus-Cell Interactions at Picomolar Concentrations

Having proven the ability of the rSAM-NPs to interact tightly with IAV we were curious to know whether this translated into an effective multivalent inhibition of IAV-cell interactions. We, therefore, focused on the well-established hemagglutination inhibition assay (HAI) (Figure 6). For this purpose, different concentrations of rSAM- and SAM-NPs were preincubated with 8 HAU of the deactivated IAV for 1 h. Thereafter the samples were exposed to glutaraldehyde-fixed turkey red blood cells (RBCs) and incubated for 30 min. In presence of the SAM- and rSAM-NPs formed from E2 only (χ_E4‑SA_ = 0) or E4-Gal with identical nominal composition as the rSAM formed from E4-SA (χ_E4‑Gal_ = 0.15), effective hemagglutination was observed in all samples indicating here a lack of inhibition (Figure 6c, Figure S10a–c). This contrasted with the samples treated with SA-containing rSAM-NPs (χ_E4‑SA_ = 0.15) (Figure 6; Figure S10d). Here effective hemagglutination was absent even at sub-pM concentrations of the NPs. Hence, the rSAM-NPs can compete with the interaction of the viral particles with the RBCs and completely inhibit their hemagglutination at very low concentrations. As the hemagglutination of the RBCs proceeds via interaction of the HA with sialic acid on the cell surface, the results again confirm that the binding of H5N1 by the rSAM-NPs indeed proceeds through the interaction between E4-SA and HA.

**Figure 6:**
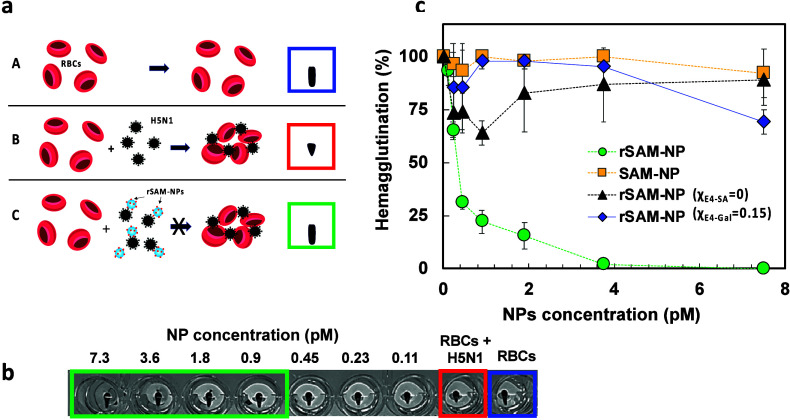
Hemgglutination inhibition assay using NP inhibitors showing (a) principle of the hemagglutination inhibition assay: (A) glutaraldehyde stabilized turkey red blood cells (RBCs) freely settling to form a teardrop shaped spot, (B) hemagglutination of RBCs by H5N1 to form a circular spot, and (C) inhibition of hemagglutination leading to a teardrop shaped spot. (b) Image of well plate after tilting showing the RBC spot appearance in the absence and presence of virus and incremental additions of rSAM-NP. Teardrop shaped spots were read as negative, whereas quasi-circular spots indicate positive agglutinated cells. (c) Degree of hemagglutination estimated for the inhibition assay of RBCs by H5N1 preincubated with SAM-NPs (orange squares), rSAM-NPs (χ_E4‑SA_ = 0) (black triangles), and rSAM-NPs (χ_E4‑SA_ = 0.15) (green circles) or (χ_E4‑Gal_ = 0.15) (blue diamonds) for 1 h before the addition of RBCs. The RBCs treated with H5N1 and the RBCs in PBS were used as a positive and negative control, respectively. Values are expressed as means ± SD, *n* = 2.

To relate these findings to previously reported multivalent SA-based inhibitors, inhibition potency is best compared on the basis of the weight concentration of inhibitor or on a per SA-basis, the latter reflecting the enhanced binding due to multivalency. From Figure 6c we estimate the lowest concentration for complete inhibition to ca. 3.5 pM, which corresponds to a weight concentration of the inhibitor of 2.7 μg/mL. The number of SAs per NP has been estimated in Table S4 assuming a monodisperse distribution of particles, each particle being a sphere with a diameter of 50 nm and covered with densely packed SAM shells. For the rSAM-NP this leads to an estimated number of SA-ligands per particle of 6638 translating into a minimum inhibitory concentration of the NP per SA basis of ca. 23 nM. Collectively, the above estimated inhibitor constants demonstrate an exceptionally efficient inhibition, comparable to previously reported inhibitors (Table 2) based on membrane covered NPs^28^ (entries 4, 5) or scaffolds featuring precisely spaced glycans (entry 3)^19^ and drastically superior to similarly sized inhibitors based on covalently linked SA-ligands (entries 1, 2).^16,17^

**Table 2:** Comparison of Synthetic SA-Based Inhibitors of RBC-IAV Interactions

					*K*_i_ ^a^		
entry	IAV	scaffold	size (nm)	sialic acids/NP	(μg/mL)	(nM sialic acid)	ref
**1**	H3N2	Polyglycerol nanogel (nPG)	50	10000	46	5.8 × 10^4^	^16^
**2**	H3N2	Hyperbranched polyglycerol (hPG)	3	35	1447	2.2 × 10^6^	^16^
**3**	H3N2	Phage capsid	25	180	^c^	234	^19^
**4**	H3N2	RBC membrane vesicle (RBCm)	232	200^b^	42	8.4	^28^
**5**	H1N1	RBC membrane vesicle (RBCm)	232	200^b^	23	4.8	^28^
**6**	H5N1	AuNP-SAM-rSAM (rSAM-NP)	50	6638	2.7	23	^d^

a Inhibition constant expressed as the lowest concentration needed for complete inhibition (Phage capsid, RBCm, rSAM-NP) or as IC50 values (nPG, hPG), in weight concentration of inhibitor or molar concentration of sialic acid ligands.

b Concentration of sialic acids expressed in nmol/mg RBCm.

c Only molar concentration reported = 1.3 nM.

d This work.

## Conclusions

The principle of multivalent recognition is being increasingly exploited in the design of synthetic receptors, mostly bioinspired by the way cells, pathogens, and proteins interact with each other.^1,4,7^ This has led drug discovery practice to move beyond small molecule inhibitor design based on the “lock and key” approach to now also comprise supramolecular multivalent inhibitors and drugs.^11,18,47,48^ These typically rely on scaffolds with appended ligands with precisely tuned density and tether designs.^19^ There is now a growing realization of the crucial role of this fine-tuning to achieve superselective binding and efficient tissue targeted medicines.^47,48^ Also crucial in this context is to master the control of ligand mobility, a parameter known to strongly affect binding affinity, selectivity, and cell signaling events,^5,9,10,49^ but practical scaffolds in this regard are few. To fill this gap, we have here introduced rSAMs as a versatile plug and play approach to design core–shell nanoparticles featuring mobile ligands. We show that our previous demonstration of the crucial role of rSAM ligand lateral diffusivity on lectin affinity^30^ also holds true for 3D designs, here in the form of sialic acid-rSAM coated AuNPs. This boosts affinity for lectins and IAV as reflected in highly sensitive optical sensing of hemagglutinin (LoD < 2 nM) and deactivated H5N1 (LoD < 1 HAU). As a logical extension of sensing applications we have tested rSAM-NPs as hemagluttination inhibitors to probe their ability to inhibit virus cell interactions. Despite a simple monosaccharide ligand design we noted a highly potent inhibition with a low nanomolar inhibitory concentration per ligand unit. This is in the same range as obtained for inhibitor designs based on membrane coated nanoparticles and superior to most other reports on multivalent IAV inhibitors which feature significantly lower potencies.^18–20,50^ Apart from the enhanced binding and inhibition performance ascribed to the rSAM shell, the solid gold core offers additional translational benefits. The rapidly growing use of AuNPs as multifunctional drug delivery vehicles and diagnostic probes leverages their unique physicochemical properties in terms of well controlled and tunable synthetic protocols, straightforward conjugation chemistry, optically responsive properties, and low toxicities.^51^ The latter was confirmed by our preliminary assessment of cytotoxicity of the rSAM-NPs exposed to a human lung cancer cell line, which indicated a high cell viability and downregulation of inflammatory signals.

In summary, the core–shell architecture presented here leads to bioactive NPs displaying exceptionally high affinity for lectins and virus-like particles. By introducing several orthogonally binding ligands, we are currently designing rSAMs that not only can bind but also discriminate between closely related species.^32^

## Methods

### Preparation of SAM Modified AuNPs (SAM-NPs)

The functionalization of the citrate stabilized nanoparticles was performed via a ligand-exchange reaction. A solution of the MUA-TEG (0.15 mM, 500 μL) in dry ethanol was added dropwise to 3 mL of AuNP suspensions (60 pM in deionized water, pH 11) under vigorous stirring. After thiol addition, the suspension gradually changed from red to bordeaux. The reaction was allowed to proceed for 72 h, whereafter the colloidal solutions were centrifuged (8000 rpm, 10 min) and the pellet redispersed in (HEPES) buffer (HB) (10 mM, pH 8, 0.005% (w/w) Tween 20) followed by repeating the centrifugation and redispersion. The centrifugation-redispersion cycles were repeated five times.

### Preparation of rSAM Modified SAM-NPs (rSAM-NPs)

Stock solutions of the amidines E2 and E4-SA were prepared by dissolving them (*c* = 2.5 mM) in HEPES buffer (10 mM, pH 8). E2 or the mixture of E2 and E4-SA (χ_E4‑SA_ = 0.15 or χ_E4‑SA_ = 0.20) or E4-Gal (χ_E4‑Gal_ = 0.15) stock solutions (2.4 μL) were added to 1 mL of the SAM-NP suspension (60 pM) in HEPES buffer (10 mM), under shaking, resulting in a final amidine concentration of 6 μM in the reaction mixture. Immediately after the addition of amidines, the suspension turned purple followed by sedimentation of the nanoparticle agglomerates. After 72 h, the rSAM-modified NP colloidal solutions were centrifuged (8000 rpm, 10 min) and the pellet redispersed in HEPES buffer (10 mM, pH 8, 0.005% Tween20) followed by repeating the centrifugation and redispersion at least three times. Galactose functionalized rSAM-NPs were prepared in an identical manner by replacing E4-SA with E4-Gal.

### Characterization of rSAM-NPs by MALDI-ToF Mass Spectrometry

A matrix solution was prepared by dissolving 2,5-dihydroxybenzoic acid (DHB) (25 mg) in 1 mL of MeCN/water (50/50 v/v) containing 1% phosphoric acid and 0.1% TFA. 0.5 μL portion of the matrix solution and 0.5 μL of the NP colloidal solution (8.4 nM in HEPES buffer) were deposited on the target plate and mixed. Mass-spectrometric analysis of the samples was performed by using a MALDI reflector time-of-flight mass spectrometer (Ultraflex mass spectrometer, Bruker-Daltonics GmbH, Bremen, Germany) equipped with a Scout-384 source in positive reflector mode. The spectra were collected by accumulating 5000 laser shots in the linear mode (relative laser focus: 80%) and further analyzed with Flexanalysis 3.0 software (Bruker Daltonics).

### Characterization of rSAMs by *in Situ* Ellipsometry (ISE)

The adsorption process of amidines was monitored by using *in situ* null ellipsometry. The instrument used was an automated Rudolph thin film ellipsometer (type 43603–200E, Rudolph Research, USA) using an angle of incidence of 68°. The light source was a xenon lamp, filtered to λ = 442.9 nm. The thiol-SAM modified gold substrates (vide supra) were immersed vertically into an ellipsometric quartz cuvette with ordinary microscopic cover glass windows containing 5 mL of HEPES buffer (10 mM, pH 8.0). The cuvette was thermostated to 25 °C and equipped with a magnetic stirrer at a constant stirring rate of 350 rpm. Before each measurement, the refractive index of the SAM-modified gold substrate was determined by a 4-zone surface calibration. After a stable baseline was obtained, 100 μL of stock solution containing amidines E2 and E4-SA (χ_E4‑SA_ = 0.15; 2.5 mM) was added to the cuvette, resulting in a final total amidine concentration of 50 μM. Kinetics data were collected until stabilization. The system was then rinsed with pH 8 HEPES buffer for 300 s (11 mL min^–1^) in a continuous system. The thickness and the adsorbed mass were calculated using a three-layer substrate/film model assuming the refractive index of the liquid to be 1.335. The effective complex refractive index for the rSAMs was assumed to be 1.45. A refractive index increment, d*n*/d*c*, of 0.22 mg/mL was used to determine the amount of rSAMs adsorbed.

### Interactions between Proteins and NPs Studied by UV–vis Spectroscopy and DLS

Incremental volumes of HA or ConA stock solutions (4.2 μM in PBS, pH 7.4) were added to the NP colloidal solutions (300 μL, 0.5 nM) in PB to make up protein concentrations of 1 nM, 2 nM, 4 nM, 6 nM, 8 nM, and 12 nM. After 1 h, the samples were analyzed by UV–vis spectroscopy and DLS. The position of the plasmon peak was determined as an average of three independent experiments and the peak shift was calculated by fitting the plasmon peak with the pseudo-Voigt function using at least 40 data points.

### Inhibition of HA-rSAM-NP Interaction in the Presence of Mucin

A mucin solution (25 μL, 0.25 mg/mL, PB buffer) was added to an equal volume of HA solution (25 μL, 144 nM, PB buffer). The mixture was incubated for 10 min at room temperature under agitation and then added to 250 μL of rSAM-NPs in PB buffer. The final concentrations of HA and rSAM-NPs in the sample were 12 nM and 30 pM respectively. After 30 min, the samples were analyzed by UV–vis spectroscopy (Tecan Trading, Switzerland) and DLS (Zetasizer Ultra, Malvern Panalytical, UK). The position of the plasmon peak was measured in three independent experiments, and the peak shift was calculated by fitting the plasmon peak with the pseudo-Voigt function by using at least 40 data points.

### Interaction between Inactivated H5N1 and NPs Studied by UV–vis Spectroscopy and DLS

Incremental amounts of BPL-inactivated influenza A virus, A/Anhui/01/2005­(H5N1)-PR8-IBCDC-RG6 (128 HAU) was added to the NP colloidal solutions (30 pM) in PB to make up 300 μL solutions containing 0.43–12.8 HAU of virus (Table S7). After 1 h, the samples were analyzed by UV–vis spectroscopy and DLS. The shift in the LSPR peak position was calculated by fitting the plasmon peak with the pseudo-Voigt function using at least 40 data points.

### Interaction of HA with Au Nanoparticles, Nanorods, and Nanocubes

Interaction of HA with MUA-TEG modified spherical Au nanoparticles (NPs), nanorods (NRs), and nanocubes (NCs) functionalized with rSAM (χ_E4‑SA_ = 0.15) was evaluated using a commercial ELISA kit for influenza H5N1 HA. 75 μL of an HA solution (30 pM in HEPES buffer) was added to 75 μL particle suspensions of different concentrations (Figure S8). After 1 h of incubation at room temperature under shaking, the colloidal solutions were centrifuged (8000 rpm, 10 min), and the supernatants were collected. The residual HA concentration in the supernatant was analyzed by ELISA according to the protocol provided by the manufacturer. The assay was based on duplicate measurements repeated three times.

### Hemagglutination Assay and Hemagglutination Inhibition Assay

Hemagglutination (HA) and inhibition of hemagglutination (HAI) assays were carried out as described in the WHO manual on influenza diagnosis and surveillance, with a few modifications. Both assays made use of glutaraldehyde-stabilized red blood cells (RBCs) (5%). The cells were rinsed with PBS buffer supplemented with 1 mg/mL BSA (PBS-BSA) three times and diluted to 0.5% suspension with PBS-BSA buffer prior to use.

#### Hemagglutination Assay

A serial dilution of virus (BPL-inactivated influenza A virus, A/Anhui/01/2005­(H5N1)-PR8-IBCDC-RG6 (128 HAU) in allantoic liquid) was prepared across rows 1–11 in a V-bottom shaped 96-well microtiter plate as follows. 50 μL of PBS (pH 7.4) was added to each of wells 1–12 and 50 μL of virus to well 1 leading to a 2-fold dilution. Then, 50 μL of virus was transferred from well 1 to well 2 leading to an overall 4-fold dilution. The procedure was repeated 11 times to complete the dilution series. 50 μL of the RBCs (vide supra) were added to each well of the plate. The solutions were mixed and incubated at room temperature for 30 min. The hemagglutination titer was then estimated as the reciprocal of the dilution factor in the last well with complete hemeagglutination. For the BPL-inactivated influenza, A H5N1Virus/Anhui/01/2005­(H5N1)-PR8-IBCDC-RG6 the HA titer was found to equal 128 hemagglutination units (HAU) (Figure S9). The assay was performed in duplicate.

#### Hemagglutination Inhibition Assay

NP solutions (0.113–30 pM) were first prepared by serial dilution of NP stock solutions (30 or 60 pM in PB) with PB. To 50 μL of each solution and a solution lacking NPs (positive control), 50 μL of virus (BPL-inactivated influenza A H5N1 Virus/Anhui/01/2005­(H5N1)-PR8-IBCDC-RG6) in allantoic liquid diluted to 4 HAU with PBS was added and the suspensions incubated under shaking for 1 h. Thereafter, 50 μL of each solution was transferred to the wells of a V-bottom shaped 96-well microtiter plate, followed by the addition of 50 μl of 0.5% glutaraldehyde stabilized turkey red blood cells (RBCs) to each well of the plate. The RBCs alone incubated with 50 μL of PB buffer were used as a negative control. The solutions were mixed and incubated at room temperature for 30 min. The plate was thereafter tilted to 90° for 30 s and then photographed. To distinguish between agglutinated and nonagglutinated cells, the shape of the RBC spots was recorded. Teardrop-shaped spots were read as negative, whereas the absence of teardrop formation indicated positive agglutinated cells. Each assay was performed in duplicate.

To obtain the degree of hemagglutination, we analyzed the path length of the RBCs after the plate was tilted using ImageJ software. We define the degree of hemagglutination as

$$h=\frac{L-\mathrm{Lc}}{\mathrm{Lv}-\mathrm{Lc}}\times 100$$

where Lc is the path length of the RBC negative control, Lv is the path length of RBC incubated with H5N1 as positive control, and *L* is the path length of the samples containing H5N1 preincubated with the NP solutions. The samples exhibiting hemagglutination exceeding 75% were assigned as fully agglutinated. The inhibitor constant *K*_i_(HAI) was defined as the lowest inhibitor concentration resulting in complete inhibition of hemeagglutination caused by the virus. The inhibitor weight (*c*_NPw_) concentration was calculated from its molar (*c*_NP_) concentration as follows:

$$c_{NPw}=c_{NP}\times N_{A}\times m_{NP}$$

$$m_{NP}=V_{NP}\times \rho =\frac{4}{3}\times \pi \times r^{3}\times \rho$$

where *N*_A_ is Avogadro’s number, m_NP_ and *V*_NP_ the weight and volume of one AuNP, and *r* and ρ the radius and density of the AuNPs.

## Supplementary Material



## Notes

Reagents, apparatus and methods and associated tables and figures (PDF)

## #

PYC Therapeutics, 6 Verdun Street, Nedlands, WA 6009, Australia
