# Supplementary material for: Gold Nanoparticles with Adaptable Self-Assembled Monolayer Shells Allow Multivalent Inhibition and Sensing of Influenza Virus at Ultralow Concentrations
Source: ACS Cent Sci. 2025 Aug 7;11(9):1659–69. doi: 10.1021/acscentsci.5c00602 (PMC12464756; doi:10.1021/acscentsci.5c00602)
Supplement: Supplementary file 1 [file oc5c00602_si_001.pdf]

Supplementary Information

Gold nanoparticles with adaptable self-assembled monolayer shells allow multi-valent inhibition and sensing of influenza virus at ultra-low concentrations

Yulia Sergeeva,* Sing Yee Yeung,[a] Thomas Hix-Janssens, Börje Sellergren*

Department of Biomedical Sciences and Biofilms-Research Center for Biointerfaces (BRCB), Faculty of Health and Society, Malmö University, 205 06 Malmö, Sweden.

a) Present address: PYC Therapeutics, 6 Verdun Street, Nedlands, WA 6009, Australia

Table of contents

1. Materials and Methods 2
2. Characterisation of rSAMs by infrared spectroscopy 5
3. Supporting tables 7
4. Supporting figures 12
5. References 20

1. Materials and Methods

Materials

Sodium hydroxide (≥98% pure), 4-(2-hydroxyethyl)-1-piperazineethanesulfonic acid (HEPES) (≥99.5% pure), Phosphate-Buffered Saline buffer (pH 7.4), and gold nanoparticles (AuNPs) (50 nm and 100 nm diameter, OD1, stabilized suspension in 0.1 mM PBS, reactant-free), and Concanavalin A (ConA), potassium phosphate monobasic (≥99.0% pure), sodium phosphate dibasic (≥99.0% pure) and mucin from porcine stomach (Type III, bound sialic acid 0.5 – 1.5 %, partially purified) were purchased from Sigma-Aldrich. Gold nanorods (NRs, *dxl* 10x45 nm) and gold nanocubes (NCs, L = 100 nm) were purchased from Nanopartz™ (USA). The ELISA kit for Influenza A H5N1 (A/Anhui/1/2005) Hemagglutinin / HA Protein (His-tag) was purchased from Sino Biological Inc (China). 2-(2-[2-(11-Mercaptoundecyloxy)-tetra-ethoxy]) acetic acid (MUA-TEG) was purchased from ProChimia Surfaces (Poland). Tween 20 molecular biology grade was purchased from AppliChem GmbH (Germany). Glutaraldehyde stabilized turkey red blood cells were purchased from antibodies-online GmbH (Germany). The phosphate buffer (10 mM, pH 7.4, 0.001% Tween-20) (PB) was prepared using potassium phosphate monobasic (0.24 g, 1.8 mM) and sodium phosphate dibasic (1.44 g, 10.1 mM). Water used for the preparation of all solutions was purified with the PURELAB® Chorus purification system (Veolia Water Technologies, Saint-Maurice, France). Influenza A (H5N1) Surveillance Antigen, BPL-Inactivated Influenza A Virus, A/Anhui/01/2005(H5N1)-PR8-IBCDC-RG6, FR-918, was generously provided through the Influenza Reagent Resource, Influenza Division, WHO Collaborating Center for Surveillance, Epidemiology and Control of Influenza, Centers for Disease Control and Prevention, Atlanta, GA, USA, and was used without further treatment. SARS-CoV-2 hexapro spike protein (prepared 2021) was kindly provided by SciLifeLab (Stockholm, Sweden). The V-bottom 96-well microtiter plates were purchased from Sarstedt (Sweden). The amidines E2, E4-Gal and E4-SA were synthesized as previously reported.[1]

Methods

Infrared Spectroscopy

Infrared reflection absorption spectroscopy (IRAS) measurements were made using a NEXUS 6400 FT-IR spectrometer (ThermoFisher Scientific, Waltham, USA), equipped with a liquid nitrogen-cooled MCT-A detector, operating from 4000-500 cm^{-1} at a resolution of 4 cm^{-1}. The

instrument was purged with compressed purified air before and during measurements using a zero-air generator. rSAMs on the planar gold substrates were characterised using a Smart SAGA™ accessory operating at an angle of incidence of 80° using an unreacted plasma cleaned gold substrate as reference. The rSAM-NPs were characterised using a Smart iTR Attenuated Total Reflectance (ATR) Sampling Accessory equipped with a diamond ATR crystal. One drop of concentrated NP dispersion was placed on the diamond ATR crystal and allowed to dry in air. Each spectrum was the sum of 500 scans and was processed and baseline corrected using the Omnic software.

Dynamic Light Scattering (DLS)

Dynamic light scattering (DLS) measurements were carried out using a Zetasizer Ultra (Malvern Panalytical, UK). Before the measurements the samples were equilibrated at 25°C for 1 min. The autocorrelation functions were analyzed using ZS XPLORER 1.0.0.436 software (Malvern Panalytical) using multiple narrow mode. The changes in NP size are mean values based on at least three independent experiments.

UV-Vis measurements

UV-Vis spectra were recorded using a Safire plate reader (Tecan Trading, Switzerland) operating in the top mode at 25 °C. The experiments were carried out at room temperature and the spectra were recorded from 300 to 800 nm. The peak positions were calculated by fitting the plasmon peak with the pseudoVoigt function using at least 40 data points.

Transmission electron microscopy (TEM)

Samples containing NPs (30 pM) and virus (12.8 HAU) were prepared as described under the interaction studies below. 10 µL of NP and virus suspension were transferred by pipette to a carbon-coated copper TEM grid (400 mesh). After 10 min at room temperature, the liquid was blotted with filter paper and a droplet of 2 wt% uranyl acetate solution was loaded onto the grid for negative staining. After 10 min the excess solution was blotted with filter paper and the grid subsequently air-dried. TEM images were obtained with an FEI Tecnai Biotwin 120 kV transmission electron microscope (FEI, USA). For a given particle, the shell thickness was estimated as the average of three measurements performed in different shell areas. The overall shell thickness was then estimated as an average shell thickness based on 10 different particles.

High performance liquid chromatography (HPLC)

The HPLC system consisted of an Agilent HPLC 1100 series instrument equipped with a UV-dual wavelength detector and an autosampler. The column was a reversed phase (C18) column (Phenomenex Luna C-18, 150 Å~ 4.6 mm), the flow rate: 0.9 mL/min, the injection volume: 10 µL and the detection performed by UV absorbance at 265 nm. The elution was conducted in the gradient mode using as mobile phase A: MeCN (0.1% TFA) and B: water (0.1% TFA) and a gradient of 10-90% A (0-8 min), 90% A (8-12min) and 10% A (12-15min). The resulting peaks eluting at 7.2 min (E4-SA) and 8.1 min (E2) were integrated for estimating the amount of bound amidine to the NPs.

Preparation of SAMs on planar gold substrates

The gold surfaces used for characterizing the rSAMs by ISE and IRAS were prepared by electron beam (e-beam) evaporation of gold (2000 Å thickness) onto precleaned glass slides (76 x 26 x 1 mm) containing adhesive layers (25 Å) of titanium. Prior to thiol adsorption, these gold surfaces were treated with plasma cleaner (PDC-32G, Harrick Plasma, USA) for 5 min at high RF power. The SAMs were prepared by immersing freshly plasma treated gold substrates in 0.15 mM MUA-TEG in ethanol (99.5%) for 24 hrs followed by rinsing with copious amount of ethanol and drying under a nitrogen stream.

2. Characterisation of rSAMs by infrared spectroscopy

IRAS characterization of rSAM-modified planar surfaces (Fig. S1 and Table S1)

The structure and composition of the films were investigated by Infrared Reflection Absorption Spectroscopy (IRAS). The spectra of the mixed rSAMs (Fig. S1) were compared with respect to features informative of amphiphile stoichiometry as well as order and orientation of the molecules. The significant peaks of the anchor SAM (MUA-TEG) and the two component rSAMs as we reported previously could all be identified (Table S1). This comprised the C-H stretch bands of the alkyl chains at 2918-2925 and 2848-2852 cm^{-1}, the sharp and intense aromatic C=C stretch signals of the bolaamphiphiles (1614, 1513, 1497, 1472 cm^{-1} on the SAM), the C-O-C stretch at ca 1265 cm^{-1}, the aliphatic ether band at 1132 cm^{-1} (MUA-TEG) and the weak C-H out of plane bending signal at 842 cm^{-1}. The intense C=C stretch bands having transition dipole vectors oriented along the 1,4-axis of the benzene rings relative to the bands representing perpendicular transitions at 842 cm^{-1} and ca 1680 cm^{-1} are in line with our previous reports and indicate a near upright position of the layer amphiphiles.[2]

Buffer stability of the films was then assessed by incubating the rSAMs in phosphate buffer (10 mM, pH 7.4, 0.005% w/w Tween 20) for 1h under shaking (Fig. S1). The intensity of the C=C stretch band at 1614 cm^{-1}, that in view of the strong 1,4-dipole can be mainly ascribed to the benzamidine head group,[3] appeared only weakly affected. This suggests that the rSAM remain stable upon the phosphate buffer treatment. Other subtle changes of the spectra were observed in the form of a pronounced increase of the signals at 2919, 1497, 1196 and 842 cm^{-1} featuring transition dipole vectors orthogonal to the 1,4-axis. Ruling out presence of adsorbed buffer components as a cause for these intensifications, we attribute these changes to a more tilted arrangement of the amidine amphiphiles, notably involving the middle benzene group and the alkane chain of the molecules.

Characterisation of rSAM modified Au-NPs (Fig. S4 and Table S5)

Fig. S4 shows the normalized attenuated total reflection infrared spectra (ATR-IR) of the SAM- and rSAM-NPs deposited on the ATR crystal from a pH 8 buffered solution. The SAM-NP spectrum is dominated by bands ascribable to the outer tetraethylenglycol (TEG) -carboxylate segment of MUA-TEG. Based on previous extensive characterisation of PEG-based protein resistant surfaces most of these bands can be assigned with a high degree of confidence and used to derive structural information with respect to chain order and conformation.[4] The first

feature that stands out is the strong band at 3397 cm^{-1} originating from hydrated carboxylates of the ionized SAM, an assignement supported by the broad COO^- stretch band at 1667 cm^{-1}.[5] The reason why this band appears here and not in the spectra of the SAM on planar gold (Fig. S1) is connected to the sample preparation. The NPs were deposited from a pH 8 dispersion with the head groups mainly ionized whereas the planar gold samples were recorded after sorption of the carboxylic acid thiols in the protonated form from an ethanolic solution. The 3397-band disappeared after rSAM modification being replaced by OH(NH)-stretching bands at 3484 and 3534 cm^{-1}. These can originate from the amidine anchor or head group of the filler amidine E2 or from free COOH groups. The appearance of a C=O stretch signal at 1727 cm^{-1} supports here the latter explanation. These new features indicate that the amidinium-amphiphiles effectively neutralize the anionic head groups, a result in line with the strongly reduced negative charge of the particles (Table 1).

The high frequency region reveals other structural details in the CH-stretching region 2700-3000 cm^{-1}. The 2934 cm^{-1} band is indicative of an amorphous arrangement of the TEG chains whereas the shoulder bands at 2918 and 2848 cm^{-1} point to the presence of well-ordered alkyl chains of the anchor SAM. In the low frequency region, bands at 1620, 1273 and 838 cm-1 reasonably well match the rSAM spectral signature but unfortunately no unambiguous assignement is here possible due to contributions from the underlying SAM. More interesting is to study the TEG characteristic C-O-C ether stretching bands showing maxima at 1186 for the SAM and 1178 cm^{-1} for the rSAM. This broad intense band is composed of several overlapping vibrations with positions and widths informative of the EG chain conformation.[4] Common for the SAM and rSAM-NPs is the somewhat weaker band at 1126 cm^{-1} with a shoulder at lower frequencies. This has been assigned to a C-O-C stretching mode of helical oligoethyleneglycol chains with the low frequency contribution (1114 cm^{-1}) reflecting crystallinity. The position of this band is close to the dominating band of the SAMs on planar gold indicating a higher degree of order in the latter chains. This contrasts with the NPs featuring band maxima at higher frequencies possibly indicating chains in a disordered state. Interestingly a pronounced shift to lower frequencies (from 1186 to 1178 cm^{-1}) of this band maximum occurs upon rSAM modification suggesting a major influence of the latter on the EG segments (TEG and EG tethers of E2 and E4-SA) conformation and order.

3. Supporting tables

Table S1. Positions and spectral mode assignments of the IR bands of a mixed rSAM of E2 and E4-SA ($\chi_{E4\text{-}SA}$=0.15) formed in Hepes buffer (pH 8) on an anchor SAM of MUA-TEG on gold and subjected to incubation in PB (10 mM, pH 7.4, 0.005 w/w% Tween 20) for 1h. Modes highlighted in red refer to functionalities present in the sialic acid head group.

**Mode assignment**	**Wavenumbers ν (cm^{-1})**	
	SAM	rSAM
NH_2, N-H stretch; COOH, O-H stretch, CONH, N-H stretch (trans)	-	3218 b
Benzene, C–H stretch	-	3000
Alkyl, CH_2, C–H stretch (asym)	2922	2919
Alkyl, CH_2, C–H stretch (sym)	2848	2848
Amidinium, N-C=N stretch (asym)<br>COOH, CONH, C=O stretch	-	1688 b
COO^- stretch (asym)	1630 b	-
Aromatic C=C stretch (ǁ1,4 axis)	-	1614/1513/1497/1471
COO^- stretch (sym), COH deform	-	-
Aromatic ethers, aryl-O-CH_2- stretch (asym)	-	1265
COOH, C-O stretch<br>Aromatic C-H bending (in plane)	-	1196
Aliphatic ethers, C-O-C (asym)	1132	1132
Aromatic C–H bending (out of plane)	-	842

b: broad; sh: shoulder; w: weak; s: strong.

Table S2. Composition, plasmon band positions and average size by DLS (D_h) of 50 nm AuNPs modified with MUA-TEG and rSAM of E2 and E4-SA ($\chi_{E4\text{-}SA}$=0.15) in HEPES buffer (10 mM, pH 8, 0.005 w/w% Tween 20).

Name	Au-NP size D (nm)	Plasmon band position (λ,nm)	Estimated D_h (nm)
Au-NP_{100}	100	570±1	109±1
SAM-NP_{100}	100	574±0.2	113±1
rSAM-NP_{100}	100	575±1	114±1
Au-NP	50	533±2	67±2
SAM-NP	50	538±0.3	71±1
rSAM-NP	50	540±0.4	73±1

Table S3. Estimation of rSAM-NP shell composition based on HPLC quantification of unbound amphiphiles

Property	E4-SA		E2		Total amidines	
	Nominal value	Measured value	Nominal value	Measured value	Nominal value	Measured value
**HPLC peak area**	4.4	3.9±0.2	27	34.8±0.3	-	-
**C_0 (µM)**	0.9	-	5.1	-	6	-
**C_f (µM)**	0.6	0.5	3.3	4.2	3.9	4.7
**C_b (µM)**	0.3	0.4	1.8	0.9	2.1	1.3
**n_b (nmol)**	2.6	3.3	14.8	7.1	17.4	10.4
**χ (mol/mol)**	0.15	0.3	0.85	0.7	1	1
**n_b/n_b^{nom} x100 (%)**	100	119	100	47	100	58

A suspension of AuNPs (60 pM) containing E2 and E4-SA ($\chi_{E4\text{-}SA}$ = 0.15) with a total concentration (C_0) of 6 µM was incubated as described above by shaking for 72h followed by centrifugation and separation of the supernatant. Quantification of the residual free (C_f) and bound (C_b) concentration of amphiphiles was subsequently performed by reversed phase HPLC in order to estimate the shell composition with respect to the nominal values. The results are averages of two independent experiments.

Table S4. Estimation of the number of thiols and amidines bound to one nanoparticle

Number and composition per NP	Nominal value	Measured value
**Number of thiols**	36682	-
**Number of amidines**	36682	21275 ± 733
**Number of E4-SA**[a]	5502	6638 ± 326

a) Theoretical number of SA units per particle was estimated using equation 1.

Eq. 1: $SA_{\#}/NP = \chi_{E4SA} A_{NP}/A_{amph}$, with $A_{NP} = area\ of\ one\ NP = \pi D^2$ and $A_{amph} = area\ occupied\ by\ one\ amphiphile = 22\ \text{Å}^2$ assuming a densely packed monolayer.[6]

Table S5. Positions and spectral mode assignments of IR bands of MUA-TEG SAM-NPs before and after modification with rSAM of E2 and E4-SA ($\chi_{E4\text{-}SA}$=0.15). Modes highlighted in red refer to functionalities present in the sialic acid head group.

Mode assignments	SAM-NP ν (cm^{-1})	rSAM-NP ν (cm^{-1})
Alcohol, free COOH, O-H stretch; NH_2, N-H stretch, CONH, N-H stretch	-	3534, 3484
COO^-, hydrated (b, h-bonded)	3397 b [5]	-
TEG, CH_2, C-H stretch (asym, amorph)	2934	2932 [4a]
Alkyl, CH_2, C-H stretch (asym)	2918 sh	2917
TEG, CH_2, C-H stretch (sym)	2884	2884 sh [4b]
Alkyl, CH_2, C-H stretch (sym)	2848 sh	2848
OH, NH combination band	2700-2830	2700-2830
COOH, C=O stretch<br>Amidinium, N-C=N stretch (asym)	-	1727
COO^- stretch (**a**sym)<br>Amidine, N-C=N stretch (asym)	1667 b	1647 b, sh
Aromatic, C=C stretch	-	1620 b
TEG, CH_2, C-H bending	1463	1463, 1446 sh
TEG, CH_2, C-H bending (amorph)	1352, 1315	1352, 1311
Aryl ethers, C-O-C- stretch (asym)	-	1273
TEG, C–O–C stretch (asym)	1229 s, 1193 s, 1186 s, 1126 s	1207 s, 1187 s, 1178 s, 1126 s
TEG, CH_2, C-H bend	1055 s, 1043 s, 1004 s	1055 s, 1043 s, 1004 s
Aromatic C–H bending (out of plane)	832	838

b: broad; sh: shoulder; w: weak; s: strong.

Table S6. Properties of proteins used to assess NP affinity and selectivity.

	Hemagglutinin (HA)	Concanavalin A (ConA)
Mw (Da)	62 000[a]	102 000[b]
Dimensions (Å)	135x (15-40) x (15-40)[c]	42 x 40 x 39[d]
pI	6.0 - 7.8	4.5 – 5.5
Ligand	α-2,3 or α-2,6-linked sialic acid	α-mannose/α-glucose

a: Molecular weight of the monomeric form

b: Molecular weight of the homotetrameric form

c: Dimension of the homotrimeric form

d: Dimension of one subunit

Table S7. Volume of the BPL-inactivated influenza A virus, A/Anhui/01/2005(H5N1)-PR8-IBCDC-RG6 (128 HAU) added to the NP suspensions and the corresponding final concentrations of the virus in the samples. The total sample volume was kept at 300 uL.

Volume of the H5N1 in allantoic liquid, μL	Virus concentration in the sample, HAU
0	0
1	0.43
2	0.86
4	1.72
6	2.58
8	3.44
12	5.16
16	6.88
22	9.46
30	12.8

4. Supporting figures

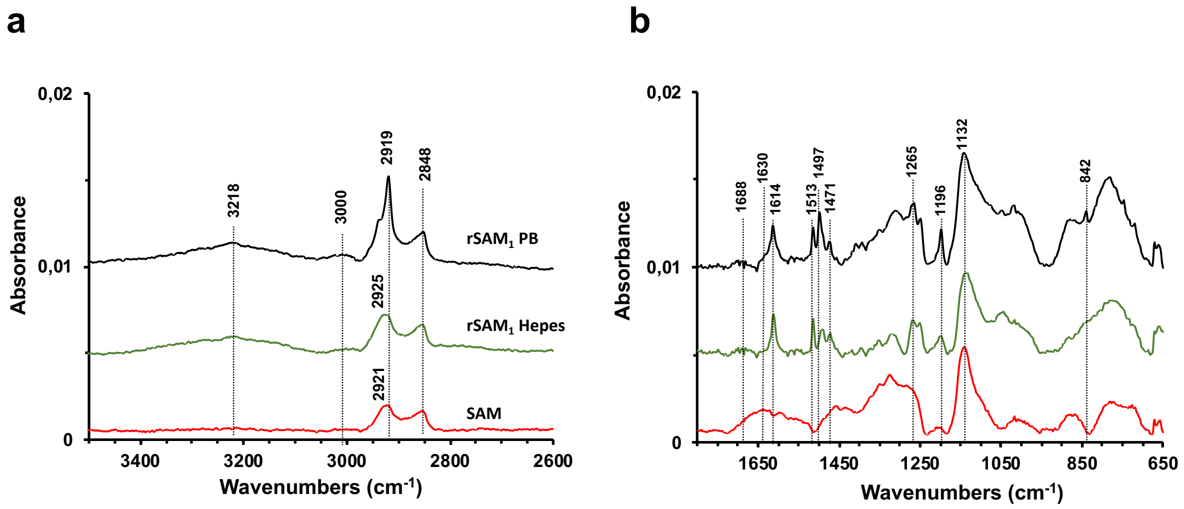

Figure S1. Baseline-corrected high (a) and low (b) frequency regions of IRAS spectra of rSAM$_1$ ($\chi_{E4\text{-}SA}$ = 0.15) (black and green spectra) on the SAM (lower red spectra) of MUA-TEG after assembly in HEPES buffer (10 mM, pH 8) (green spectra) and after subsequent 1h incubation in PB buffer (10 mM, pH 7.4, 0.005% Tween) (upper black spectra).

a **b**

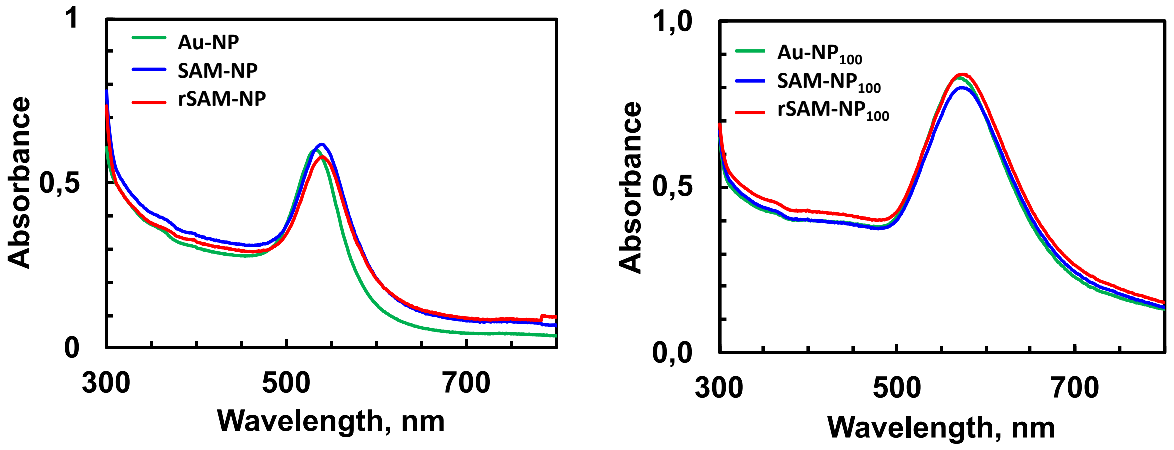

Figure S2. Typical UV-Vis spectra of 50 nm (a) and 100 nm (b) AuNPs modified with MUA-TEG and rSAM ($\chi_{E4\text{-}SA}$=0.15).

a

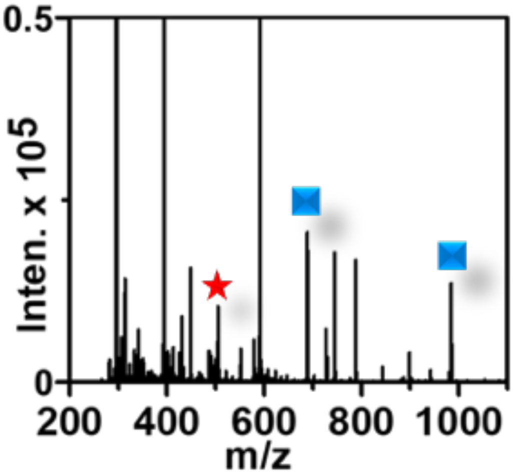

Assignment	Signal (m/z)
E4-SA	975, 684
E2	501
MUA-TEG	295, 394 (-COOH), 591 (dimer)

b

c

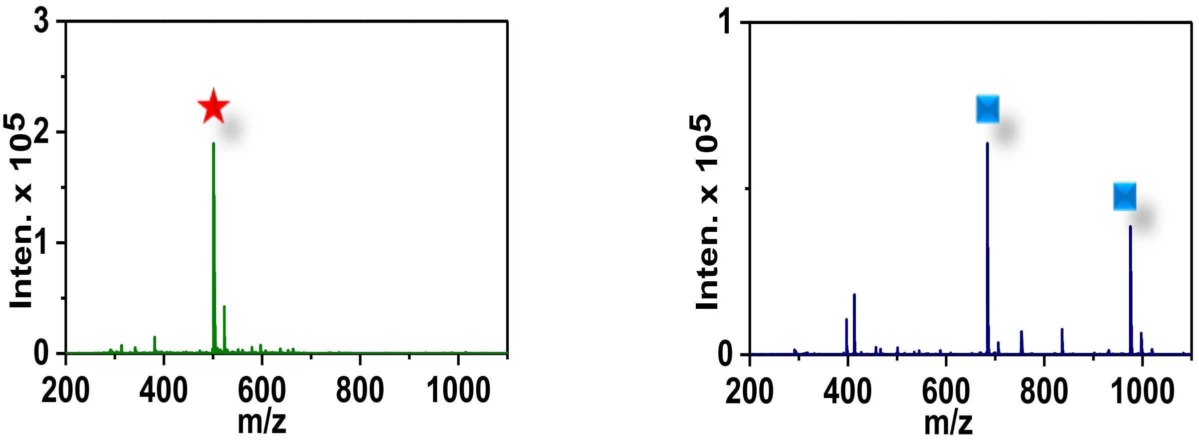

Figure S3. MALDI-TOF mass spectrum recorded for a) rSAM-NPs (8.4 nM, HEPES buffer, pH 8), b) E2 and c) E4-SA and the corresponding signal assignements.

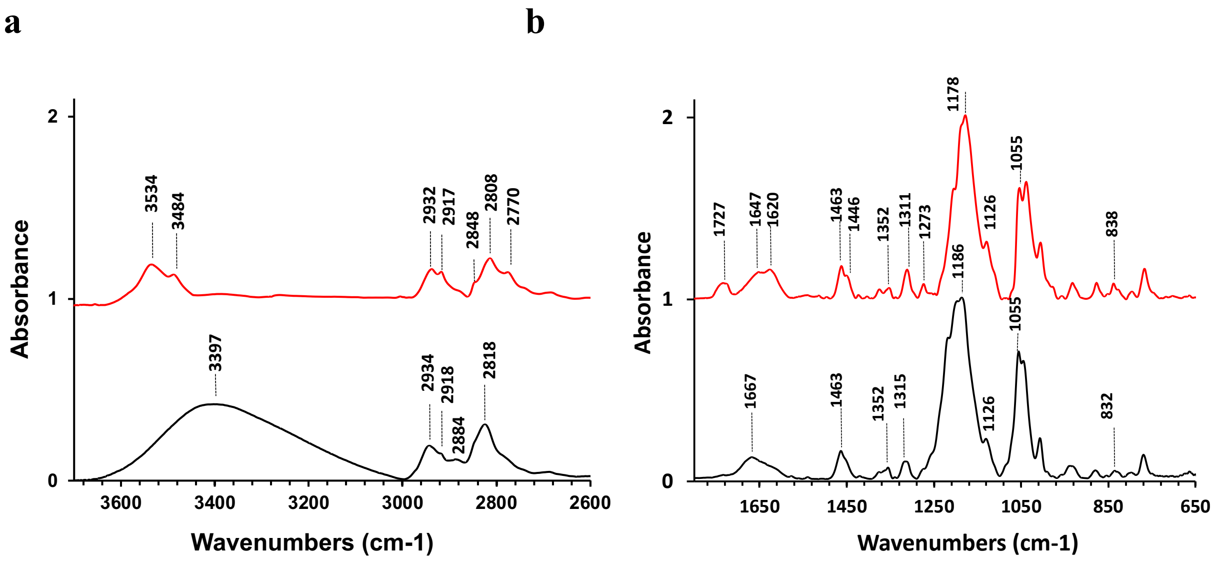

Figure S4. Normalized baseline-corrected high (a) and low (b) frequency regions of IR-ATR spectra of SAM-NPs (lower black spectrum) and rSAM-NPs ($\chi_{E4\text{-}SA}$ = 0.15) (upper red spectrum).

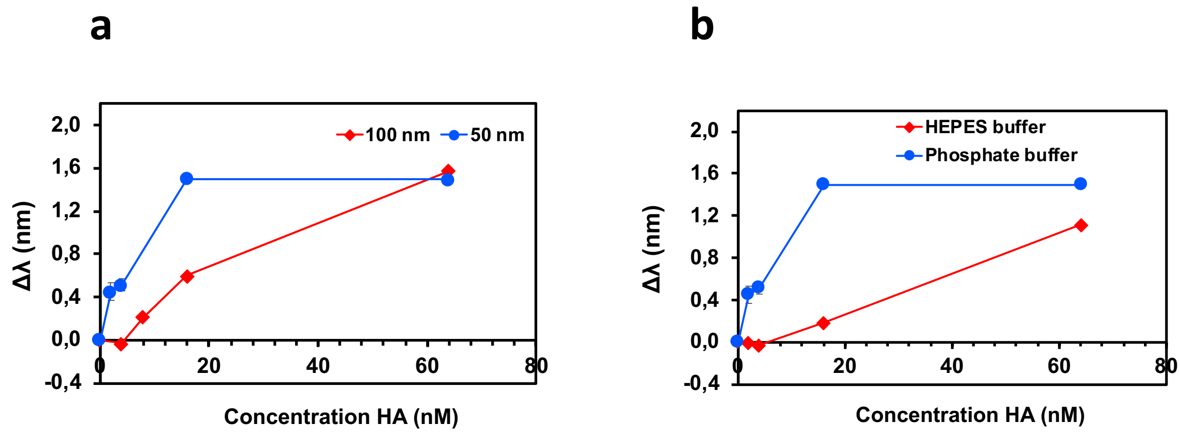

Figure S5. Optimization of the rSAM-NP based LSPR assay. Comparison of the red shift of NPs in response to increasing HA concentration for a) 50nm and 100nm NPs in phosphate buffer (10 mM, pH 7.4, 0.005% Tween) and b) 50 nm NPs in HEPES buffer (10mM, pH 8) or phosphate buffer (10 mM, pH 7.4, 0.005% Tween).

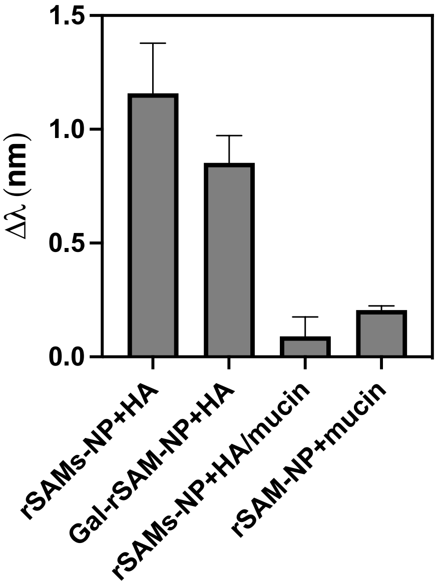

Figure S6. Comparison of plasmon band shift of rSAM-NPs in response to HA (12 nM), mucin (0.02 mg/mL) or HA preincubated with mucin in phosphate buffer (10 mM, pH 7.4, 0.005% Tween20). Values are expressed as means ±SD, n=3.

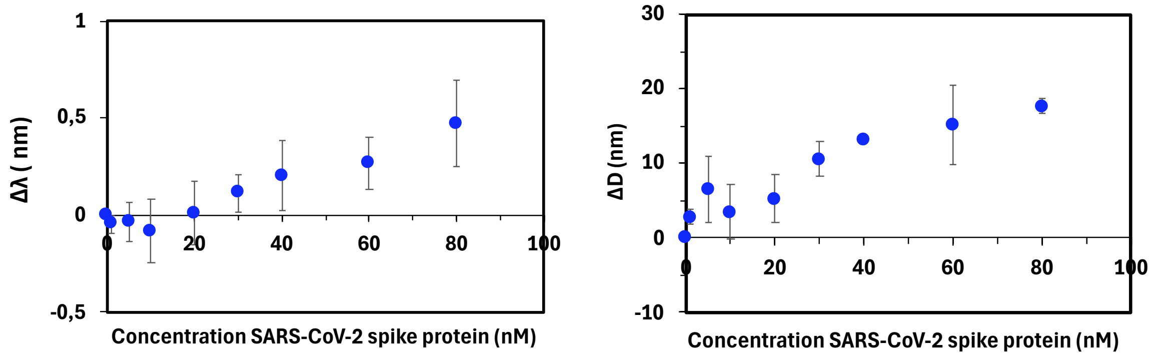

Figure S7. (a) Plasmon band shift of the rSAM-NPs (cNP= 30 pM; χE4-SA=0.15) as a function of SARS-CoV2 spike protein concentration in phosphate buffer (10 mM, pH 7.4, 0.005% Tween20)) and (b) the parallel increase in the hydrodynamic diameter of the NPs. Values are expressed as means ±SD, n=3

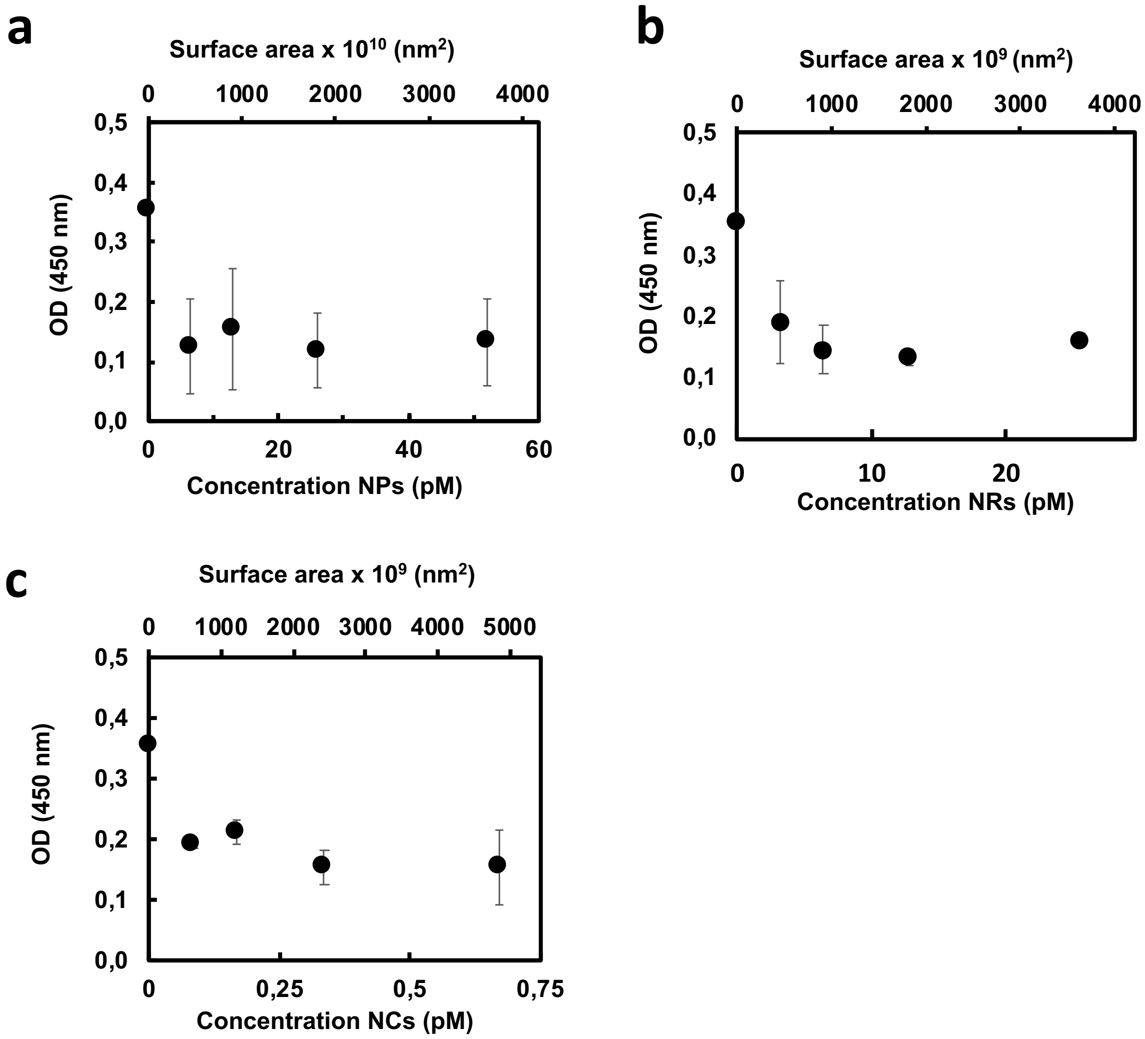

Figure S8. ELISA detection of residual HA in the supernatant after incubation with spherical nanoparticles (NPs, a), nanorods (NRs, b), and nanocubes (NCs, c) functionalized with rSAMs ($\chi_{E4\text{-}SA}$=0.15). The initial HA concentration was 30 pM. For b) and c) the concentration of the nanoparticles was adjusted to yield the same total surface area. All values are expressed as means ±SD, n=3.

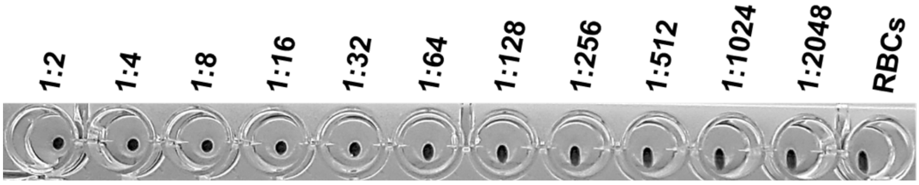

Figure S9. Hemagglutination assay for BPL-Inactivated Influenza A Virus, A/Anhui/01/2005(H5N1)-PR8-IBCDC-RG6 using glutaraldehyde stabilized turkey red blood cells. The HAU titer based on the transition of the spots from circular to teardrop shape was estimated to 128.

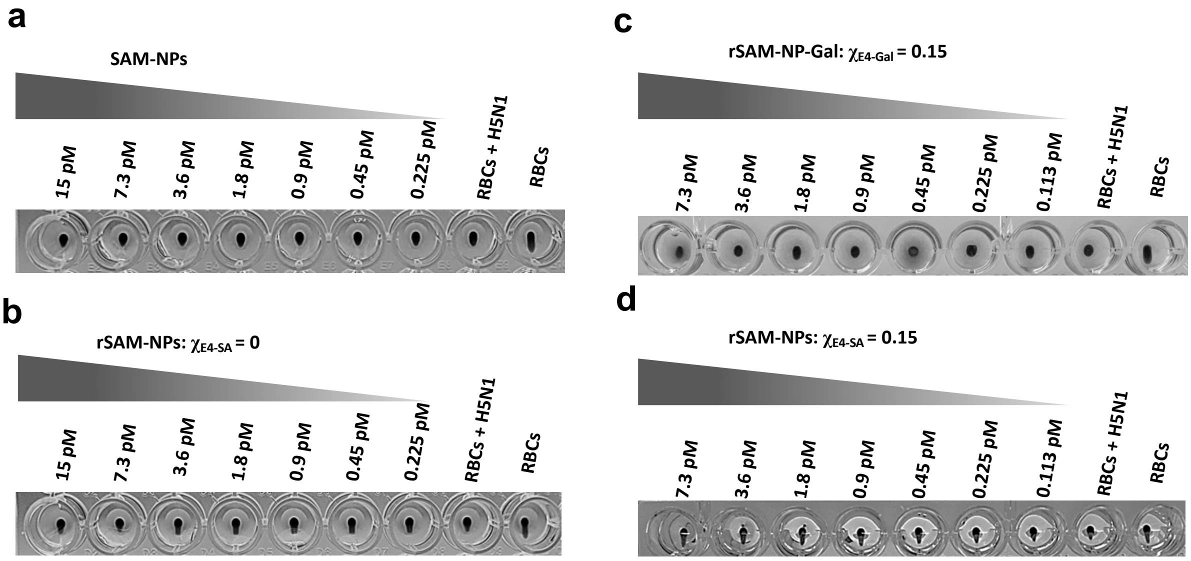

Figure S10. Hemagglutination of glutaraldehyde stabilized turkey red blood cells by H5N1 pre-incubated with SAM-NPs (a) and with rSAM-NPs with (b) $\chi_{E4\text{-}SA}$=0 and (c) $\chi_{E4\text{-}Gal}$ = 0.15 and (d) (c) $\chi_{E4\text{-}SA}$=0.15 for 1 h before the addition of RBCs. The RBCs treated with H5N1 and the RBCs in PBS were used as positive and negative controls, respectively. The assays were performed in duplicate.

5. References

[1] a) S. Y. Yeung, Y. Sergeeva, T. Dam, P. Jonsson, G. Pan, V. Chaturvedi, B. Sellergren, *Langmuir* **2019**, *35*, 8174-8181; b) Y. Sergeeva, S. Y. Yeung, B. Sellergren, *ACS Applied Materials & Interfaces* **2024**, *16*, 3139-3146.

[2] F. Auer, G. Nelles, B. Sellergren, *Chemistry – A European Journal* **2004**, *10*, 3232-3240.

[3] S. D. Evans, E. Urankar, A. Ulman, N. Ferris, *J. Am. Chem. Soc.* **1991**, *113*, 4121-4131.

[4] aP. Harder, M. Grunze, R. Dahint, G. M. Whitesides, P. E. Laibinis, *J. Phys. Chem. B* **1998**, *102*, 426-436; bR. Valiokas, S. Svedhem, S. C. T. Svensson, B. Liedberg, *Langmuir* **1999**, *15*, 3390-3394.

[5] J.-J. Max, C. Chapados, *The Journal of Physical Chemistry A* **2004**, *108*, 3324-3337.

[6] J. C. Love, L. A. Estroff, J. K. Kriebel, R. G. Nuzzo, G. M. Whitesides, *Chemical Reviews* **2005**, *105*, 1103-1170.
